# Pathophysiology, clinical presentation, and treatment of coma and acute kidney injury complicating falciparum malaria

**DOI:** 10.1097/QCO.0000000000000419

**Published:** 2018-01-04

**Authors:** Katherine Plewes, Gareth D.H. Turner, Arjen M. Dondorp

**Affiliations:** a Faculty of Tropical Medicine, Mahidol Oxford Tropical Medicine Research Unit, Mahidol University, Bangkok, Thailand; b Division of Infectious Diseases, Department of Medicine, University of British Columbia, Vancouver, British Columbia, Canada; c Department of Cellular Pathology, John Radcliffe Hospital; d Nuffield Department of Clinical Medicine, Center for Tropical Medicine and Global Health, University of Oxford, Oxford, UK

**Keywords:** cerebral malaria, malaria-associated acute kidney injury, pathophysiology, treatment

## Abstract

**Purpose of review:**

Cerebral impairment and acute kidney injury (AKI) are independent predictors of mortality in both adults and children with severe falciparum malaria. In this review, we present recent advances in understanding the pathophysiology, clinical features, and management of these complications of severe malaria, and discuss future areas of research.

**Recent findings:**

Cerebral malaria and AKI are serious and well recognized complications of severe malaria. Common pathophysiological pathways include impaired microcirculation, due to sequestration of parasitized erythrocytes, systemic inflammatory responses, and endothelial activation. Recent MRI studies show significant brain swelling in both adults and children with evidence of posterior reversible encephalopathy syndrome-like syndrome although targeted interventions including mannitol and dexamethasone are not beneficial. Recent work shows association of cell-free hemoglobin oxidation stress involved in the pathophysiology of AKI in both adults and children. Paracetamol protected renal function likely by inhibiting cell-free-mediated oxidative stress. It is unclear if heme-mediated endothelial activation or oxidative stress is involved in cerebral malaria.

**Summary:**

The direct causes of cerebral and kidney dysfunction remain incompletely understood. Optimal treatment involves prompt diagnosis and effective antimalarial treatment with artesunate. Renal replacement therapy reduces mortality in AKI but delayed diagnosis is an issue.

## INTRODUCTION

**FIGURE 1:**
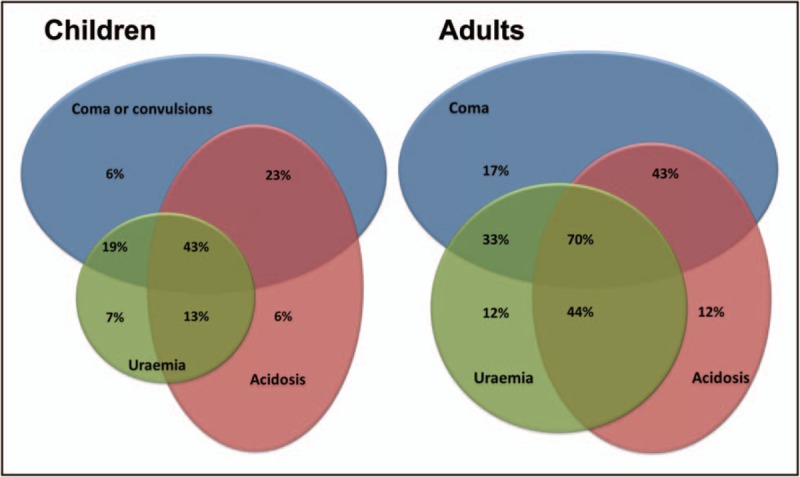
Venn diagrams of mortality of adults and children associated with prognostic manifestations of severe malaria [2,3]. Surface areas represent relative prevalence in severe malaria. Uremia defined as blood urea nitrogen more than 20 mg/dl in children and more than 48 mg/dl in adults. Acidosis defined as base excess less than −8 mmol/l in children and less than −3 mmol/l in adults. Coma score defined as Blantyre Coma Score less than 3 in children and Glasgow Coma Score less than 11 in adults. Reprinted from Tropical Medicine and International Health 19, Supplement 1, World Health Organization, Severe Malaria. Page 16. Copyright (2014).

Severe malaria incidence is approximately two million cases with nearly 430,000 deaths annually [1]. It is a medical emergency characterized by multisystem disease with different clinical manifestations between adults and children. However, recent studies show that cerebral involvement, kidney dysfunction, and acidosis are independent predictors of mortality in both adults and children (Fig. 1) [2,3]. This is supported by a meta-analysis of children with severe malaria that found prognostic indicators with the strongest association with death to be acute kidney injury (AKI) (odds ratio 5.96, 95% confidence interval, CI: 2.9–12.11) and coma (4.83, 95% CI: 3.11–7.5) [4^▪▪^].

Cerebral malaria is a clinical syndrome of impaired consciousness associated with malaria in the absence of hypoglycemia, convulsions, drugs, and nonmalarial causes characterized by unrousable coma defined by a Glasgow Coma Score less than11 (adults) [5] or Blantyre Coma Score less than 3 (children) [6–8]. Two large intervention trials in Asian adults and African children with severe malaria found that 54% of adult and 34% of pediatric patients had cerebral malaria [9,10]. AKI in severe falciparum malaria is caused by acute tubular necrosis and defined as a creatinine more than 265 μmol/l or urea more than 20 mmol/l [6]. In adults with severe malaria, AKI develops in up to 40% of patients, whereas in children, the incidence is historically reported at approximately 10% [9,10]. As the WHO definition does not define AKI adequately for pediatric malaria, the reported incidence of AKI in children is likely underestimated. The recent Kidney Disease: Improving Global Outcomes (KDIGO) classification standardizes AKI for clinical practice and research [11]. In adult severe malaria, 58% had AKI as defined by KDIGO, of whom 40% died, accounting for 71% of overall mortality [12^▪^]. Among children with severe malaria 46% had AKI as defined by KDIGO, of whom 12–24% died with increasingly severe AKI [13^▪^]. In two large multicenter studies, approximately 25% of children with severe malaria had increased blood urea nitrogen, accounting for roughly 50% of total deaths [10,14]. These studies imply that AKI complicating pediatric severe malaria has been previously underdiagnosed [13^▪^].

Notably, the majority of patients surviving these complications have complete recovery after appropriate treatment. The direct cause of coma and AKI complicating severe malaria are incompletely understood but likely share common pathophysiological mechanisms. This review will highlight recent developments in our understanding of the pathophysiologic and pathologic processes associated with cerebral malaria and malaria-associated AKI in addition to the clinical presentation, diagnosis and treatments of these complications. 

**Box 1:**
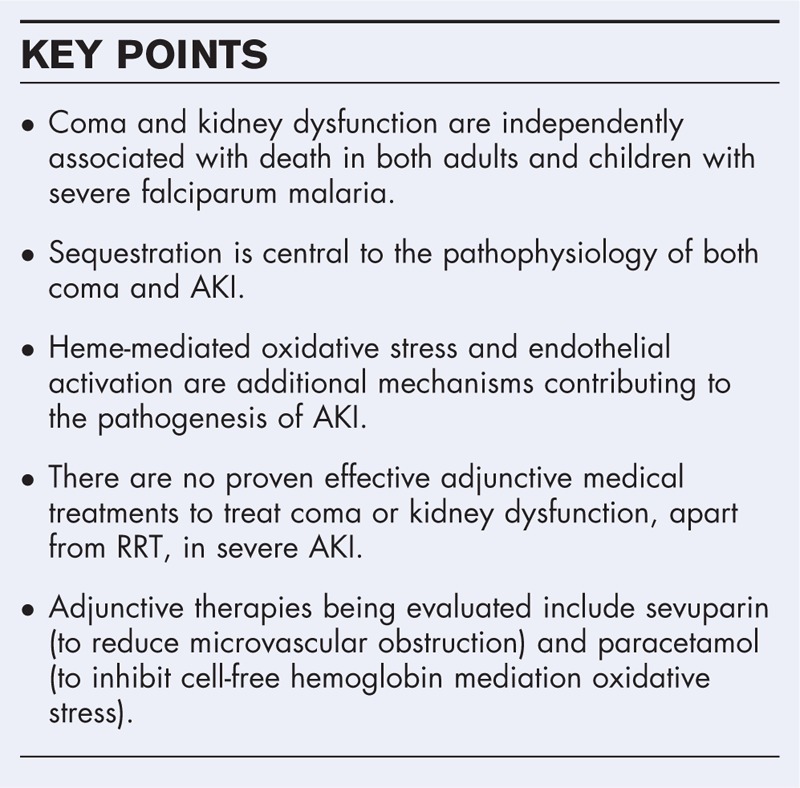
no caption available

## PATHOGENESIS

Severe malaria is predominantly caused by *Plasmodium falciparum* because of its ability to induce infected red blood cell (RBC) cytoadherence to the vascular endothelium and consequent end-organ dysfunction. Other plasmodium species can cause severe disease and AKI [15], although their ability to cause coma is debated [6].

### Microvascular obstruction

Parasites developing within the infected RBC transport *P. falciparum* erythrocyte membrane protein 1 (*Pf*EMP1) to the RBC membrane functioning as a key ligand for cytoadherence [16]. *Pf*EMP1 is expressed on RBC protrusions, or ‘knobs’, that confer points of attachment to the endothelium. *Pf*EMP1 is strain specific, encoded by a highly variable *var* gene family, which provides antigenic variation for immune evasion and differential endothelial receptor binding. CD36 is an endothelial receptor constitutively expressed on most vascular beds [17]. The key endothelial receptor in the brain is intercellular adhesion molecule-1 [18]. Recent studies have identified endothelial protein C receptor as an important receptor in the brain that binds to a specific *Pf*EMP1 domain (CIDRα1) [19^▪^,^20^–^22^]. Cytoadhesion results in sequestration of parasitized RBCs in the capillaries and postcapillary venules causing heterogeneous blockage of the microcirculation and tissue hypoxia [23]. In addition to flow obstruction by sequestered parasitized RBCs, microcirculatory flow is thought to be further compromised by increased rigidity of both infected and uninfected RBCs and clumping of infected RBCs (platelet-mediated autoagglutination) and uninfected RBCs adhering to infected RBCs (rosette formation) [24].

Direct visualization of microvascular obstruction is observed in the retina of adults and children with cerebral malaria, termed ‘malaria retinopathy’ [25,26]. Cerebral blood flow is not decreased in adults [27,28]. Intracranial pressure is often increased in children, but less so in adult patients [29,30]. Indirect assessment of sequestration via estimated total parasite biomass, measured by *P. falciparum* histidine rich protein 2, was shown to contribute to AKI in adults with severe malaria [31]. Autopsy studies of adults and retinopathy-positive children dying from cerebral malaria show prominent sequestration in the brain microvasculature compared to adults with fatal noncerebral malaria and retinopathy-negative children [32,33]. Postmortem studies report sequestration of parasitized RBCs in renal glomerular and peritubular capillaries in adults and children [32,34].

### Endothelial activation

Microvascular obstruction-induced tissue hypoxia is compounded by microvascular dysfunction [35,36] and increased oxygen demand [36,37]. In adults with cerebral malaria, there is endothelial and astroglial activation in the brain [18], with variable inflammatory responses [38] and mild functional change to the blood–brain barrier [39,40]. In children with strictly defined retinopathy-positive cerebral malaria, breakdown of the endothelial barrier is observed particularly in areas of sequestration [32]. Patterns of histopathological change within the brain in cerebral malaria vary between adults and children, with less inflammatory cellular infiltrates and edema in adult cases [41,42]. A recent pediatric autopsy study found that HIV coinfection influences histopathology, increasing the degree of platelet and monocyte infiltration around damaged microvasculature [43].

In a recent MRI study of similarly defined children, 35% had evidence of brain swelling most commonly in fatal cases implicating brainstem herniation as the cause of death [44]. Another recent serial MRI study in India including adults and children found that 50% of patients had evidence of brain swelling with posterior vasogenic edema and vascular congestion in the basal nuclei [45^▪^]. All patients had rapid clinical improvement and radiological reversibility with hallmarks suggestive of posterior reversible encephalopathy syndrome. The exact cause of the brain swelling is yet unclear.

Studies of patients with severe malaria having AKI show reduced renal cortical blood flow [46], increased kidney size [47], and endothelial changes in both glomerular and peritubular capillaries on histopathology [34]. Cell-free hemoglobin and lipid peroxidation markers are strongly associated with AKI and renal replacement therapy (RRT) requirement in adults with severe malaria [12^▪^]. In children with severe malaria, an elevated heme-to-hemopexin ratio was associated with hemoglobinuria, stage 3 AKI, and 6-month mortality [48^▪^].

### Cytokines

There is an imbalance of proinflammatory and anti-inflammatory responses in severe malaria [49]. The role of cytokines and chemokines in cerebral malaria has been recently reviewed [50]. However, many of these studies are in the murine experimental cerebral malaria model, the relevance of which has been questioned [51]. Conflicting evidence has emerged from human studies as to the association between cerebral malaria and levels of numerous cytokines such as tumor necrosis factor α (TNFα) [49,52–56]. Although cytokines and/or chemokines are clearly involved in the pathogenesis of malarial fever and may be associated with disease severity and/or cerebral malaria, it is not established that they are a cause of coma.

The role of cytokines and chemokines in the pathophysiology of AKI in severe malaria was recently highlighted. Plasma-soluble urokinase-type plasminogen activator receptor, a marker of immune activation, was independently associated with AKI and RRT requirement [31]. Previously, it was shown that TNFα, but not inteleukin (IL)6 or IL6:IL10 ratio, was associated with AKI suggesting that TNFα may induce localized renal tubular cell injury [57].

## CLINICAL FEATURES

**FIGURE 2:**
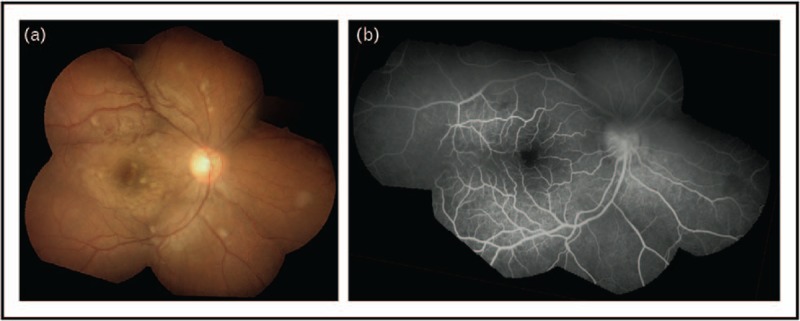
Malaria retinopathy in a Bangladeshi child with cerebral malaria. (A) Composite fundus photograph. (B) Fluorescein angiogram of same fundus image. Images show widespread retinal whitening and patchy hypoperfusion with a white-centered hemorrhage. Typical malarial retinopathy can include four findings: first, macular (perifoveal) and peripheral retinal whitening, second, retinal vessel whitening/discoloration, third, white-centered hemorrhages, and fourth, papilledema. The former two features (first and second) are specific for malaria and the latter two features (third and fourth) are also found in nonmalarial conditions. Reproduced with permission from BMJ Publishing Group Ltd [97].

The clinical presentation of cerebral malaria is diffuse symmetrical encephalopathy with fever and absent or few focal neurological signs. In children, coma can rapidly develop after fever onset (mean, 2 days) [7]. In adults, coma is typically gradual with increasing drowsiness, confusion, obtundation, and high fevers (mean duration, 5 days). Convulsions are present in approximately 15% of adults and 80% of children with severe malaria [9,10] and frequently herald development of coma. Patients may recover full consciousness after a convulsion, thus transient postictal coma must be excluded [6]. Multiple convulsions are common and up to 50% of comatose children have subclinical seizures or status epilepticus. Ocular funduscopic findings include vessel color change, macular and extramacular whitening, and white-centered retinal hemorrhages [58] (Fig. 2).

Among survivors, the median time to coma recovery is roughly 24 h in children and 48 h in adults [9,10]. Retinal abnormalities resolve with no residual visual deficit. Neurologic sequelae occur in less than 1% of adults but up to 12% of children in the quinine-therapy era, including hemiplegia, cortical blindness, aphasia, and cerebellar ataxia [59]. Studies suggest that neurologic deficits may reflect slow neurological recovery [10,60]. Postmalaria neurological syndrome is self-limiting [61]; however, longer term neurological sequelae, including cognitive deficits and epilepsy, are reported among children [62,63^▪^].

The majority of malaria patients have risk factors for developing AKI, including volume depletion, hypoalbuminemia, male sex, previous AKI, concomitant bacterial sepsis, blackwater fever (BWF), or comorbidity, such as, diabetes. Severe intravascular hemolysis and hemoglobinuria in severe malaria, with or without AKI, is known as BWF. Although oliguria clinically indicates decreased function with a prerenal component, up to 80% of patients with malaria have nonoliguric AKI [64–66]. Thus, the clinical diagnosis will capture established anuric AKI but will underdiagnose moderate AKI and delay diagnosis. AKI complicating severe malaria can be categorized into four groups:

(1) Few severity criteria with prerenal AKI that resolves with fluids.
(2) Several severity criteria including AKI that resolves without RRT.
(3) Progressive AKI that resolves with antimalarial treatment and RRT.
(4) Multiorgan dysfunction, often with anuric AKI and cerebral malaria, who die prior to or during RRT with hemodynamic shock and/or respiratory failure.

## DIAGNOSIS

Any comatose patient with a history of fever and/or travel to malaria-endemic regions must be considered to have cerebral malaria until proven otherwise. In children, febrile convulsions should be distinguished from cerebral malaria, wherein coma will persist beyond 1 h after anticonvulsive treatment is administered. Absence of fever does not rule out malaria. Antimalarial treatment should not be delayed in severely ill patients if diagnostics are unavailable or delayed.

Parasitological diagnosis is by microscopy of stained thin and thick blood smears. A rapid diagnostic test for parasite antigens can be performed if microscopy is unavailable. Patients may have a low circulating parasitemia because of sequestration, thus a low parasitemia is not reassuring [67]. In high-transmission settings, children with partial immunity tolerate higher parasitemia without severe symptoms and may be asymptomatic at low parasitemia. A parasitemia of more than 1 000 000/μl in African children with cerebral malaria is associated with fatal outcomes [7].

Funduscopy for malaria retinopathy improves specificity for diagnosis of cerebral malaria and is prognostic in patients with severe malaria [26,68,69] (Fig. 2). Alternative causes of coma must be ruled out including hypoglycemia, and bacterial, fungal, or viral meningoencephalitis. Lumbar puncture does not increase mortality in stable, comatose children with suspected cerebral malaria even when MRI brain swelling or papilledema is present [70^▪^].

**FIGURE 3:**
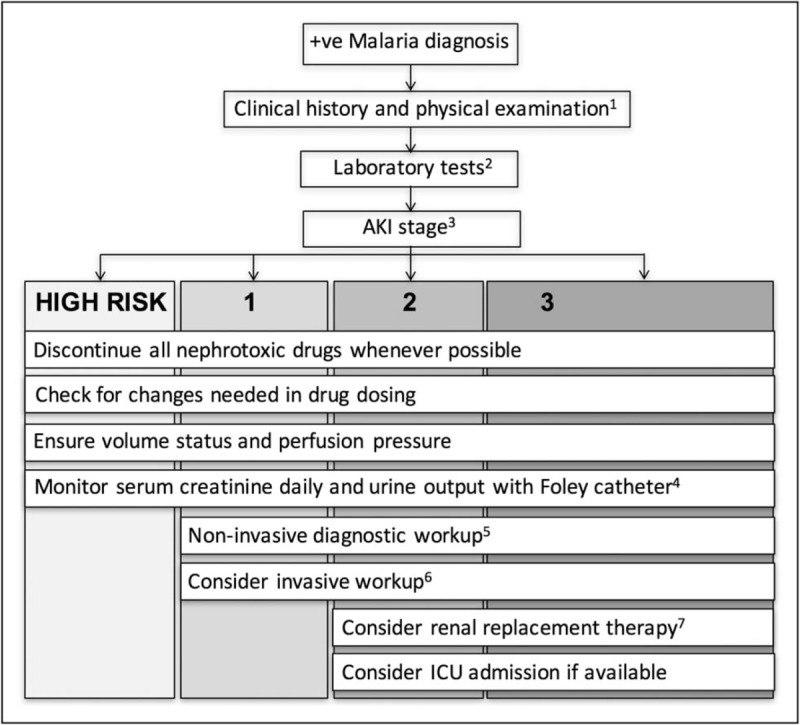
General management of AKI in malaria based on KDIGO guidelines. ^1^History including preadmission medications, comorbidities, and physical examination focusing on volume status and signs of concomitant sepsis. ^2^Laboratory tests including serum creatinine, urea, electrolytes, and full blood count. ^3^Stage using KDIGO staging criteria. If baseline creatinine is unknown, estimate using back-calculation of MDRD equation (>19 years) or Swartz equations (≤18 years). Urinary bladder catheterization to monitor initial urine output if unconscious. If ambulating, urine should be collected to monitor output. Patients should be managed according to AKI stage, as stage correlates with increased morbidity and mortality. ^4^Daily creatinine and urine output to monitor change in AKI stage severity and guide management. ^5^Additional investigations to assess AKI etiology: urine analysis, sediment microscopy, creatinine, and sodium; renal ultrasound to assess kidney size, presence of pyelonephritis, and inferior vena cava filling as a gauge of volume status; AKI biomarkers if applicable. Nephrotoxic drugs, that is, aminoglycosides, should be avoided whenever possible. Discontinuation of nephrotoxic drugs may assist with determining AKI etiology. ^6^If resources permit, monitor hemodynamic variables. Static central venous pressure is of limited value but recommended target is 0 to +5 cmH_2_O. Arterial pulse pressure as a dynamic variable may be more useful to gauge response to fluid administration. ^7^Early referral to center with RRT, particularly if one indication for RRT is present. Patients with multiorgan dysfunction are recommended to receive urgent dialysis within 2 h [6]. Patients should be evaluated 3 months after AKI resolution to monitor resolution of kidney function and/or development of chronic kidney disease. AKI, acute kidney injury; KDIGO, Kidney Disease: Improving Global Outcomes; MDRD, modification of diet in renal disease [11]; RRT, renal replacement therapy. Reprinted from Kidney International Supplements 2, Kidney Disease: Improving Global Outcomes (KDIGO) Acute Kidney Injury Work Group, KDIGO Clinical Practice Guideline for Acute Kidney Injury. Page 25. Copyright (2012) with permission from Elsevier.

There is no robust prognostic risk model or biomarker that can predict AKI or RRT requirement [71,72^▪^]. All patients with malaria should be considered at risk of developing AKI. To improve outcomes, early diagnosis and management is critical. AKI diagnosis requires quantification of creatinine (or urea) or observing low urine volume (<0.5 ml/kg/h) for 6 h (Fig. 3) [6,11]. Urine output is difficult to accurately assess in malaria endemic countries and may delay diagnosis. As the WHO creatinine threshold is not applicable to children, the diagnosis of AKI must be considered using all available patient data. The KDIGO AKI definition of a creatinine rise at least 1.5 times baseline is the current standard, and baseline creatinine can be back-calculated using the Modified Diet in Renal Disease (>19 years) or Swartz equation (≤18 years) [11,73].

## TREATMENT

The two key pillars of severe malaria treatment are prompt antimalarial treatment and supportive management. Adjunctive therapies targeted at the underlying pathophysiology are unproven.

### Antimalarial treatment

Two landmark trials in patients with severe malaria definitively showed that intravenous artesunate reduced mortality by 35 and 23% in adults and children, respectively, compared to quinine [9,10]. Intravenous artesunate is now the first-line treatment for severe malaria as recommended by the WHO. Artemether and quinine are the second-line therapies [74]. The mechanism of improved survival over quinine is the rapid cidal activity of artesunate on young ring forms, preventing parasite maturation and sequestration [75]. Once the patient is able to take oral medication, and after a minimum of 24 h of artesunate, an oral artemisinin-based combination therapy can be initiated to complete the treatment.

### Supportive treatment

Despite the best available artemisinin therapy for malaria, mortality remains unacceptably high and supportive management is key to reducing this. Comatose patients require endotracheal intubation with mechanical ventilation for airway protection. Rapid sequence intubation should be performed to prevent transient hypercapnia and increased intracranial pressure. Routine care should be implemented including regular turning, lateral positioning (‘recovery position’), and catheterization. Nasogastric tube insertion and suctioning may protect against aspiration, however, enteral feeding in nonintubated patients should be delayed (>60 h) because of increased risk of aspiration pneumonia [76].

The majority of children with cerebral malaria experience convulsions. Glucose replacement to ensure euglycemia and fever control with paracetamol are important. Prophylactic anticonvulsant therapy is not recommended. A randomized controlled trial (RCT) of phenobarbital in pediatric cerebral malaria showed increased mortality, likely caused by respiratory depression [77].

Fluid management and nephrotoxic drug avoidance are cornerstones for management of malaria-associated AKI. Cautious fluid management is important, as patients with AKI are not necessarily hypovolemic and are at high risk of developing pulmonary edema [78–81]. Rapid infusions may exacerbate intracranial hypertension and precipitate cerebral herniation. The large multicenter Fluid Expansion as Supportive Therapy study of African children with severe febrile illness showed a relative risk for death of 1.59 (95% CI: 1.10–2.31) with fluid bolus therapy among those with malaria [14]. The WHO recommends individualized restrictive fluid management, keeping the patient slightly dry, using slow infusion of isotonic crystalloids [74]. Patients with BWF require creatinine and hemoglobin monitoring as resulting severe anemia requires whole blood transfusion.

Treatment of malaria-associated AKI with RRT reduces mortality from 75 to 26% [64]. In general, RRT is urgently indicated when biochemical disturbances and volume overload refractory to conventional therapy are present. The additional thresholds included in the WHO malaria guidelines are based on findings that anuria and elevated or rapidly rising creatinine are sensitive indicators for RRT [6,64]. As AKI in malaria rapidly progresses and is often compounded by multiorgan dysfunction, early RRT is recommended. Although intermittent hemodialysis and continuous venovenous hemofiltration have been shown to be superior to peritoneal dialysis in adults with severe malaria [82], in the absence of hemodialysis, life-saving peritoneal dialysis should be initiated if this is the only modality available [83]. RRT has also been shown to be effective in the management of malaria-associated AKI in pediatric patients [84].

Many adjunctive therapies have been suggested, mainly driven by studies in murine experimental cerebral malaria. However, none has proven benefit in humans. Evidence for exchange transfusion and the more recently employed RBC exchange transfusion remains limited [85^▪^,^86^,^87^]. Mannitol [88,89], steroids [90,91], and monoclonal antibodies to TNF [54,56] are not recommended as treatments in cerebral malaria as studies show no benefits and potential harm. Furosemide and mannitol are ineffective in preventing and treating AKI and BWF, respectively, and may be harmful [92,93]. On the basis of the ability of paracetamol to inhibit hemoprotein-mediated AKI [94], a recent RCT of paracetamol in Bangladeshi adults with severe malaria found that acetaminophen improved kidney function and reduced the development of AKI, particularly in patients with high cell-free hemoglobin levels at enrollment [95^▪^,^96^]. Larger studies of paracetamol in adults and children with malaria are currently ongoing to further assess this renoprotective effect.

## CONCLUSION

Cerebral malaria and AKI complicating severe malaria are prognostic for mortality in both adults and children. Microvascular obstruction and endothelial dysfunction are common mechanisms for both of these complications. Future study of adjunctive therapies should target reducing sequestration, improving endovascular function, and reducing hemoglobin-mediated oxidative stress.

## Acknowledgements

None.

### Financial support and sponsorship

This work was supported by the Wellcome Trust of Great Britain.

### Conflicts of interest

There are no conflicts of interest.

## REFERENCES AND RECOMMENDED READING

Papers of particular interest, published within the annual period of review, have been highlighted as:

▪ of special interest
▪▪ of outstanding interest
